# Evolution in Obesity and Chronic Disease Prevention Practice in California Public Health Departments, 2010

**DOI:** 10.5888/pcd11.120177

**Published:** 2014-11-13

**Authors:** Liz Schwarte, Samantha Ngo, Rajni Banthia, George Flores, Bob Prentice, Maria Boyle, Sarah E. Samuels

**Affiliations:** Author Affiliations: Samantha Ngo, MPH/MSW candidate at the University of California, Berkeley, Berkeley, California; Rajni Banthia, Resource Development Associates, Oakland, California; George Flores, The California Endowment, Oakland, California; Bob Prentice, Partnership for the Public’s Health/Public Health Institute, Oakland, California; Maria Boyle, Abt Associates, Inc, Cambridge, Massachusetts; Sarah E. Samuels, The Sarah Samuels Center for Public Health Research and Evaluation, Oakland, California. At the time of the study, Ms Schwarte, Ms Ngo, Dr Banthia, and Ms Boyle were affiliated with The Sarah Samuels Center for Public Health Research and Evaluation, Oakland, California. Dr Prentice is now retired. Sarah E. Samuels died March 29, 2014.

## Abstract

**Introduction:**

Local health departments (LHDs) are dedicating resources and attention to preventing obesity and associated chronic diseases, thus expanding their work beyond traditional public health activities such as surveillance. This study investigated practices of local health departments in California to prevent obesity and chronic disease.

**Methods:**

We conducted a web-based survey in 2010 with leaders in California’s LHDs to obtain diverse perspectives on LHDs’ practices to prevent obesity and chronic disease. The departmental response rate for the 2010 survey was 87% (53 of California’s 61 LHDs).

**Results:**

Although staff for preventing obesity and chronic disease decreased at 59% of LHDs and stayed the same at 26% of LHDs since 2006, LHDs still contributed the same (12%) or a higher (62%) level of effort in these areas. Factors contributing to internal changes to address obesity and chronic disease prevention included momentum in the field of obesity prevention, opportunities to learn from other health departments, participation in obesity and chronic disease prevention initiatives, and flexible funding streams for chronic disease prevention. LHDs that received foundation funding or had a lead person or organizational unit coordinating or taking the lead on activities related to obesity and chronic disease prevention were more likely than other LHDs to engage in some activities related to obesity prevention.

**Conclusion:**

California LHDs are increasing the intensity and breadth of obesity and chronic disease prevention. Findings provide a benchmark from which further changes in the activities and funding sources of LHD chronic disease prevention practice may be measured.

## Introduction

Local health departments (LHDs) dedicate resources and attention to preventing obesity and chronic disease (1), thus expanding their work beyond traditional public health activities such as disease surveillance and control to increasing access to healthful food and physical activity. This work occurs particularly in lower income communities of color where obesity and chronic disease rates are highest. This approach parallels research demonstrating that environmentally focused interventions to prevent obesity and chronic disease have a greater impact than individual behavior modification approaches (2,3). LHDs can play a leadership role in promoting these interventions (4–6); however, little is known about LHDs’ capacities for expanding their work to prevent obesity and chronic disease. Given high obesity and chronic disease rates (7), growing momentum in the field of chronic disease prevention, and the fiscal crises counties face (8), it is crucial to monitor LHDs’ abilities to maintain and deepen activities for preventing obesity and chronic disease.

The Sarah Samuels Center for Public Health Research and Evaluation conducted a survey of California LHDs in 2007 (Phase I) to understand their capacity, practices, and resources for changing nutrition and physical activity environments for preventing obesity and chronic disease (5). Samuels Center conducted a second survey in 2010 (Phase II) to understand how LHD practice had changed since the Phase I survey, hypothesizing that LHD capacities had evolved to meet the increasing need for interventions to prevent obesity and chronic disease. Phase II was also designed to learn whether foundation funding and a lead person or organizational unit for preventing obesity and/or chronic disease influences LHDs’ activities to improve nutrition and physical activity environments.

California’s LHD practice has matured to encompass community and governmental collaborations, practice equity, and regional approaches. For many LHDs, obesity prevention was the gateway through which practice has expanded to address chronic disease prevention and underlying conditions that affect health. The Phase II survey was conducted to learn about LHDs’ readiness and capacity to progress along this continuum and to provide a benchmark from which to measure the evolution of public health practice for preventing obesity and chronic disease. We present Phase II survey findings.

## Methods

### Sample

We contacted 175 staff members in California’s 61 county health departments, including public health directors, health officers, nutrition managers, chronic disease prevention directors, and obesity and chronic disease prevention coordinators and invited them to participate in the Phase II survey, which was conducted in 2010. We identified potential respondents through professional associations, Internet searches, e-mails, and telephone calls. These professional groups were selected because of the pivotal role they play in LHDs in preventing obesity and chronic disease, and to obtain diverse perspectives within LHDs. The number of respondents per department ranged from 1 to 6 and was typically 1 (mode).

### Survey measures

The Samuels Center, in collaboration with the Partnership for the Public’s Health, the Public Health Institute, and The California Endowment, developed the Public Health Departments and Obesity/Chronic Disease Prevention Phase II Survey. Survey questions assessed 1) how LHDs are working to change nutrition and physical activity environments to prevent obesity and chronic disease and 2) which factors influence their activities. We refined questions from the Phase I survey to enhance their relevance to public health practice evolution and added new questions. Although the Phase I survey focused only on obesity prevention, Phase II focused also on chronic disease prevention. We adapted environmental and policy change items from the Institute of Medicine’s (IOM’s) Local Government Actions to Prevent Childhood Obesity report (9). The survey contained 24 items and took 10 minutes to complete. The Public Health Institute Institutional Review Board determined that no review was necessary for this research. We used a web-based survey tool to administer the survey.

### Data analysis

We exported data from the web-based tool into SPSS (IBM, Inc) and analysed the LHD-level data by aggregating individual respondents’ answers to each question for each department. For yes/no questions, if at least 1 respondent from each LHD answered yes, we assigned an affirmative response to the department. For scaled questions, we averaged individual responses from each department. We grouped responses by whether LHDs received philanthropic foundation grant funding and by whether LHDs had a lead person or organizational unit for preventing obesity and chronic disease.

## Results

At least 1 individual responded to the survey in 53 of California’s 61 LHDs, equaling an 87% departmental response rate. The 53 LHDs that did respond serve nearly all (99.5%) of California’s population of 37 million (10). We asked respondents to self-identify their position: 27% were program managers for nutrition or the Special Supplemental Nutrition Program for Women, Infants, and Children (WIC), 23% were public health officers, 15% were public health directors, 4% were chronic disease directors, and 2% were physical activity managers. The remaining respondents fell into the category of “other” staff (29%), including deputy directors, health promotion managers, and nutritionists.

### Organizational plans and worksite policies

Twenty-two LHDs in Phase II either had an organizational plan for preventing obesity and chronic disease or participated in a county-wide plan, compared with 17 departments in Phase I (Figure). Thirty-one LHDs had workplace nutrition policies, physical activity policies, or both for their own staff, an increase of 5 from Phase I. Forty-one LHDs also had employee benefit programs encouraging employees to engage in physical activity such as wellness programs, fitness club discounts, and walking clubs, 3 more than in Phase I.

**Figure:**
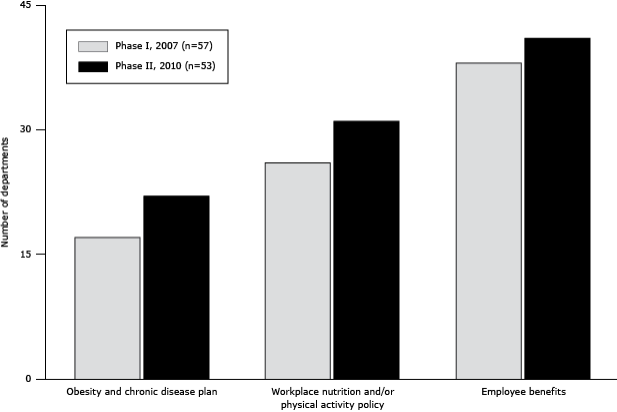
Obesity and chronic disease prevention plans and policies in local public health departments, by study phase, California Public Health Departments Obesity and Chronic Disease Prevention Survey, 2007 and 2010. **Table:** 

Plans and Policies	No. of Departments	
	Phase I, 2007 (N = 57)	Phase II, 2010 (N = 53)
Obesity and chronic disease prevention plan	17	22
Workplace nutrition and/or physical activity policy	26	31
Employee benefits	38	41

### Staff capacity

In 72% (n = 38) of LHDs, there was a lead person or organizational unit for preventing obesity and chronic disease. At those organizational units, activities included health promotion (21%), community health (16%), chronic disease prevention (16%), and health policy (3%). Although most departments either lost staff (59%) or had the same number of staff (26%) as in 2006, many departments increased their effort (62%) or put about the same effort (12%) into preventing obesity and chronic disease.

Respondents ranked their capacity (knowledge, skills, and/or expertise) for preventing obesity and chronic disease on a rating scale (1, poor/none; 2, fair; 3, good; 4, excellent). LHDs, on average, reported that they have good or excellent capacity for nutrition-related activities. They ranked providing access to healthful food, communications, community organizing, intersectoral collaboration, and leadership capacities as good. LHDs reported having fair research capacity, urban planning/community design/transportation capacity, and violence prevention capacity, and fair or poor/none legal expertise. The barriers most commonly cited for being unable to engage in activities related to preventing obesity and chronic disease were limited training (45%) and salaries too low to attract quality staff (19%).

### Strengthening focus

In the 4 years preceding the survey, 74% (n = 39) of the departments had changed internally in 1 or more ways to address obesity and chronic disease prevention. Over half (53%) of LHDs had reorganized internally; 51% had implemented nutrition and physical activity policies and programs. Nearly half of the departments had trained staff (47%) and increased leadership (45%).

Of 39 LHDs indicating internal changes, 77% attributed changes to increased momentum in the field of obesity prevention (Table 1). LHDs also attributed change to other contributors: regional and statewide efforts in preventing obesity and chronic disease (67%); learning from other health departments (59%); and obesity prevention initiatives such as Healthy Eating, Active Communities (HEAC), Central California Regional Obesity Prevention Program (CCROPP), and the public health department mini-grants program (46%).

**Table 1:** Contributions to Increased Emphasis on Obesity and Chronic Disease Prevention by California Local Public Health Departments^a^ (n = 39), 2006–2010

Contributor	No. (%) Local Health Departments
Momentum in the field of obesity prevention	30 (77)
Regional and state obesity and chronic disease prevention efforts	26 (67)
Learning from other public health departments	23 (59)
The California Endowment’s obesity and chronic disease prevention initiatives (eg, HEAC, CCROPP, Mini-grant)	18 (46)
Local and state legislation supporting obesity and chronic disease prevention	15 (39)
Technical assistance to public health departments	15 (39)
Participation in other philanthropic initiatives (eg, HEAL, RWJF, other initiative)	14 (36)
Leadership development programs or opportunities	10 (26)

Abbreviations: HEAC, Healthy Eating, Active Communities; CCROPP, Central California Regional Obesity Prevention Program; HEAL, Healthy Eating Active Living; RWJF, Robert Wood Johnson Foundation.

a Remaining local health departments surveyed either had not changed internally (n = 11) or did not know whether they had changed (n = 3).

### Funding sources

Almost two-thirds of LHDs used WIC funding for preventing obesity and chronic disease, followed by realignment (funding from the transfer of program responsibility and revenue sources for sales tax and vehicle license fees from the state to counties) (60%) and Title V (Maternal, Child, and Adolescent Health) funding (57%) (Table 2). LHDs that received foundation funding were more likely than LHDs without foundation funds to use local general funds (69%) and Network for a Healthy California funds (75%) as additional resources for preventing obesity and chronic disease.

**Table 2:** Funding Sources for Obesity and Chronic Disease Prevention Activities in California Local Public Health Departments (LHDs) (N = 53), 2010

Funding Source	No. (%) of LHDs With the Funding Source
**Governmental funding**	
**Federal**	**46 (87)**
WIC	35 (66)
Title V (MCAH)	30 (57)
Title XIX (MCAH)	18 (34)
ARRA	8 (15)
Federal Transportation Funding SAFETEA-LU	8 (15)
CDC Categorical funding	3 (6)
CDC Steps to a Healthier US	1 (2)
**State**	**43 (81)**
Realignment^a^	32 (60)
California Department of Public Health Network for a Healthy California	24 (45)
California Project LEAN	5 (9)
California Active Communities Center for Physical Activity	1 (2)
**Local general fund**	**26 (49)**
**Foundation funding**	
One or more foundation funding sources	26 (49)
Kaiser Permanente HEAL	9 (17)
Kaiser Community Health Initiative	9 (17)
HEAC	7 (13)
CCROPP	6 (11)
Healthy Kids Healthy Communities (RWJF)	3 (6)
Active Living by Design (RWJF)	2 (4)
Other foundation funding	17 (32)

Abbreviations: WIC, Special Supplemental Nutrition Program for Women, Infants, and Children; MCAH, Maternal, Child and Adolescent Health; ARRA, American Recovery and Reinvestment Act; SAFETEA-LU, Safe, Accountable, Flexible, Efficient Transportation Equity Act: a Legacy for Users; CDC, Centers for Disease Control and Prevention; LEAN, Leaders Encouraging Activity and Nutrition; HEAL, Healthy Eating Active Living; HEAC, Healthy Eating, Active Communities; CCROPP, Central California Regional Obesity Prevention Program; RWJF, Robert Wood Johnson Foundation.

a In California, “realignment” refers to funding from the transfer of program responsibility and revenue sources for sales tax and vehicle license fees from the state to counties.

### Engagement in activities

All responding LHDs engaged in at least 1 of the IOM’s recommended environmental and policy change activities to prevent childhood obesity. Whether they received foundation funds or had a lead or organizational unit for preventing obesity and chronic disease influenced how likely they were to engage in certain activities (Table 3).

**Table 3:** California Local Public Health Departments’ (LHDs’) Engagement in Obesity and Chronic Disease Prevention Activities, 2010

Activity	No. (%) of All LHDs (N = 53)	No. (%) of LHDs with Foundation Funding (n = 26)	No. (%) of LHDs Without Foundation Funding (n = 27)	No. (%) of LHDs With a Person or Unit Leading Obesity and Chronic Disease Prevention Efforts (n = 38)
**Research and data**				
Monitor obesity health indicators (eg, BMI, California Health Interview Survey)	47 (89)	24 (92)	23 (85)	35 (92)
Morbidity and mortality data linked to health equity efforts	34 (64)	19 (73)	15 (56)	27 (71)
Built environment assessments (eg, walkability assessments)	30 (57)	21 (81)	9 (33)	29 (76)
GIS mapping	27 (51)	17 (65)	10 (37)	24 (63)
CX3 assessments	25 (47)	19 (73)	6 (22)	23 (61)
Health impact assessments	14 (26)	12 (46)	2 (7)	12 (32)
**Collaboration**				
Participate in school or community obesity prevention collaboratives	46 (87)	24 (92)	22 (82)	34 (90)
Participate in city, county, or regional planning commissions or committees focused on improving built environment	44 (83)	25 (96)	19 (70)	35 (92)
Facilitate farmers market, agricultural industry, or food security program collaboration to increase access to healthful food	38 (72)	22 (85)	16 (59)	32 (84)
Participation with other public health departments in regional obesity prevention	32 (60)	22 (85)	10 (37)	26 (68)
**School health**				
Improve beverage and/or food environment	32 (60)	19 (73)	13 (48)	29 (76)
Ensure quality PE or physical activity opportunities (eg, enforce state PE requirements)	22 (42)	13 (50)	9 (33)	19 (50)
Ensure free, safe drinking water availability	16 (30)	11 (42)	5 (19)	15 (40)
**Food retail**				
Enable small store owners to carry more healthful, affordable food items	22 (42)	18 (69)	4 (15)	22 (58)
Support incentive programs or use zoning laws to attract supermarkets and grocery stores to underserved neighborhoods	16 (30)	11 (42)	5 (19)	16 (42)
Limit fast food restaurant access through policy or zoning measures	12 (23)	9 (35)	3 (11)	12 (32)
**Community food access**				
Encourage farmers markets to accept federal food program benefits (eg, WIC)	38 (72)	24 (92)	14 (52)	31 (82)
Encourage farmers markets and/or produce stand establishments	35 (66)	22 (85)	13 (48)	31 (82)
Monitor chain restaurant menu labeling and/or encourage nonchain restaurants to provide calorie information	15 (28)	12 (46)	3 (11)	14 (37)
Offer incentives (eg, recognition) to restaurants that promote more healthful options	7 (13)	7 (27)	0	7 (18)
**Public and worksite programs**				
Support worksite nutrition standards and/or physical activity guideline development and implementation	22 (42)	19 (73)	3 (11)	22 (58)
Support public venue nutrition standards and/or physical activity guideline development and implementation	22 (42)	15 (58)	7 (26)	21 (55)
**Physical activity environments**				
Support sidewalk and street crossing planning, and building to encourage walking	36 (68)	23 (89)	13 (48)	33 (87)
Develop and implement Safe Routes to School programs with schools to increase walkability and bikability	32 (60)	22 (85)	10 (37)	29 (76)
Support safe and attractive park and playground development near residential areas	28 (53)	16 (62)	12 (44)	25 (66)
Establish joint use of facilities agreements between school districts and other organizations	26 (49)	18 (69)	8 (30)	24 (63)
**Other policies or ordinances**				
Promote air quality improvements, including greenhouse gas reduction, transportation redesign	20 (38)	17 (65)	3 (11)	19 (50)
Promote tax or fee to discourage nutrient-poor foods and beverages	16 (30)	12 (46)	4 (15)	14 (37)
Inform federal food legislation (eg, Child Nutrition Reauthorization Act)	15 (28)	10 (39)	5 (19)	12 (32)
Develop local ordinances to restrict nutrient-poor mobile vending near schools and/or public playgrounds	10 (19)	8 (31)	2 (7)	9 (24)
Eliminate nutrient-poor food and beverage marketing in children’s environments	7 (13)	5 (19)	2 (7)	6 (16)

Abbreviations: BMI, body mass index; GIS, geographic information systems; CX3, Communities of Excellence; PE, physical education; WIC, Special Supplemental Nutrition Program for Women, Infants, and Children.

Over half of LHDs engaged in built environment assessment activities (57%) and geographic information system (GIS) mapping (51%). LHDs that received foundation funding were more likely than LHDs without foundation funds to conduct built environment studies (81% vs 33%), Communities of Excellence (CX3) (73% vs 22%), GIS mapping (65% vs 37%), and health impact assessments (46% vs 7%). Similarly, LHDs with a lead for preventing obesity and chronic disease were more likely than LHDs without a lead to perform built environment assessments (76% vs 7%), CX3 (61% vs 13%) assessments, and GIS mapping (63% vs 20%).

Eighty-three percent of LHDs participated in planning commissions or committees focused on improving the built environment, and 60% collaborated with other health departments in regional obesity prevention efforts. LHDs with foundation funding were more likely than LHDs without foundation funds to participate in school or community collaboratives or coalitions (92% vs 82%) or engage in preventing obesity and chronic disease through collaboration with other health departments (85% vs 37%). LHDs with a lead for preventing obesity and chronic disease were more likely than LHDs without a lead to be involved in school or community collaboratives or coalitions (90% vs 80%) and planning commissions or committees (92% vs 60%).

Forty-two percent of LHDs worked with schools to enforce state physical education requirements, and 30% worked toward ensuring free and safe drinking water availability. More LHDs with a lead for preventing obesity and chronic disease than LHDs without a lead worked to improve food and beverage environments (76% vs 20%), state physical education requirement enforcement (50% vs 20%), and drinking water availability (40% vs 7%).

Many LHDs helped small store owners carry more healthful, affordable items (42%), and limited fast food accessibility through zoning or policy measures (23%). More LHDs that received foundation funding helped store owners carry more healthful items than did LHDs without foundation funds (69% vs 15%). LHDs with a lead for preventing obesity and chronic disease were more likely to help store owners carry more healthful items (58% vs 0%), support incentive programs or use zoning laws to attract grocery stores to underserved neighborhoods (42% vs 0%), and limit fast food access (32% vs 0%) than LHDs without a lead.

To increase community food access, LHDs encouraged federal food assistance program acceptance at farmers markets (72%), monitored menu labeling in chain restaurants and/or encouraged nonchain restaurants to provide calorie information (28%), and offered incentives to restaurants that provide healthier options (13%). LHDs that received foundation funding were more likely than LHDs without foundation funds to promote food assistance program acceptance at farmers markets (92% vs 52%), farmers markets establishment (85% vs 48%), menu labeling (46% vs 11%), and incentives to restaurants that offer healthier options (27% vs 0%). LHDs with a lead for preventing obesity and chronic disease were more likely to promote food assistance program acceptance at farmers markets (82% vs 47%), farmers markets establishment (82% vs 27%), and monitoring menu labeling (37% vs 7%) than LHDs without a lead.

Forty-two percent of LHDs supported public venue and worksite nutrition standards and/or physical activity guidelines. LHDs that received foundation funding were more likely than LHDs that did not receive foundation funding to support nutrition and/or physical activity guidelines for worksites (73% vs 11%) and public venues (58% vs 26%). More LHDs with a lead than LHDs without a lead for preventing obesity and chronic disease supported nutrition and/or physical activity guidelines for worksites (58% vs 0%) and public venues (55% vs 7%).

Overall, 68% of LHDs supported sidewalk planning and building, and 60% developed and implemented Safe Routes to School programs with schools. LHDs that received foundation funding were much more likely than LHDs without foundation funds to engage in sidewalk planning and building (89% vs 48%), Safe Routes to Schools (85% vs 37%), and joint use agreements (69% vs 30%). Similarly, LHDs with a lead for preventing obesity and chronic disease collaborated on physical activity improvements at a much higher rate than LHDs without a lead; 87% of those with a lead and 20% of those without a lead worked on sidewalk planning and building. Of LHDs with a lead, 76% worked on Safe Routes to School (compared with 20% without a lead), 63% worked on joint use agreements (compared with 13% without a lead), and 66% supported park development (compared with 20% without a lead).

## Discussion

### Agents for change

The Phase II survey findings show that LHDs, in collaboration with local governments and community leaders, are agents of change for preventing obesity and chronic disease. As a new standard of practice, California LHDs are addressing the policies, systems, and environments that underpin good health-related outcomes.

LHDs made changes within their organizations for preventing obesity and chronic disease through reorganization, reallocation of resources, leadership development and staff training, and nutrition and physical activity policy implementation. An important finding is that LHDs attribute their organizational and practice changes to contextual factors such as learning from regional and state obesity and chronic disease prevention efforts, learning from other LHDs, and participating in obesity prevention initiatives. LHDs also favorably rated their staffs’ capacity in various skill areas needed for preventing obesity and chronic disease, demonstrating that departments are reshaping their organizations. These findings may explain why LHDs can report both increasing their efforts for preventing obesity and chronic disease and losing or maintaining the same staff or resources to support these activities.

LHDs relied on and used both governmental and foundation funding for preventing obesity and chronic disease. Foundation funding, which may allow LHDs more flexibility than governmental funding in how the funds are used, appears to have provided impetus for California LHDs to engage in changing nutrition and physical activity environments. These findings are consistent with findings from Phase I (5). More than 70% had a lead person or division for preventing obesity and chronic disease, which is consistently associated with pursuing environmental and policy approaches for preventing obesity and chronic disease.

### Evolution in practice

Public health practice in California changed considerably since our first survey in 2007. Key developments were the position paper issued by the California Conference of Local Health Officers (CCLHO) (11) in 2007 and regional workshops in 2008 to strengthen department capacity for preventing obesity and chronic disease. Additionally, the Bay Area Regional Health Inequities Initiative convened 11 LHDs to transform public health practice so as to change conditions that contribute to health disparities and cultivate recognition that social justice and equity are at the heart of reducing conditions that fuel the obesity and chronic disease epidemics (12).

Activities for preventing obesity and chronic disease gained visibility and momentum through HEAC and CCROPP — pioneering, multisector initiatives to change nutrition and physical activity environments in low-income, ethnically diverse communities. These programs achieved full implementation between Phase I and Phase II, and may explain the high level of LHD engagement in policy, systems, and environmental approaches to prevent obesity and chronic disease. Fourteen LHDs played major roles in the 2 initiatives (13–16).

Change in policy and systems at the state level further fueled momentum in LHDs to improve nutrition and physical activity environments in California. A Health in All Policies Task Force (convened by The California Department of Public Health) was created to foster collaboration between California state departments and to require consideration of the health impact of all proposed policies (17).

Although LHDs worked on preventing obesity and chronic disease with fewer resources and staff in an already challenging funding environment (18–20), new federal funding sources and sustained leadership from the public health profession have bolstered efforts. Under the federal American Recovery and Reinvestment Act of 2009, the California Department of Public Health and 3 LHDs received Communities Putting Prevention to Work (CPPW) cooperative agreements to reduce chronic disease through changes in policy, systems, and environment (21). At the time the survey was conducted, CPPW resources had not yet been awarded in California. Anticipation of these resources may have stimulated LHDs’ interest and activity in community-level chronic disease prevention and influenced survey responses. The Affordable Care Act supported Community Transformation Grants (CTGs) (22), which were awarded to 9 California LHDs to address tobacco-free living, active living and healthful eating, and clinical and other preventive services. A state CTG awarded to the Public Health Institute in collaboration with the California Department of Public Health covers implementation in 42 counties, with 12 LHDs receiving direct funding. CPPW and CTGs built on an LHD and community collaboration model catalyzed by the Partnership for the Public’s Health (23) and expanded on by HEAC and CCROPP to address policy and environmental change for obesity prevention across sectors.

In concert with the influx of CPPW and CTG resources into California LHDs, 10 LHDs representing 64% of the state’s population formed the Public Health Alliance of Southern California (formerly Southern California Chronic Disease Collaborative) in 2012 to address chronic disease prevention regionally through multisector policy, environmental, and systems change (T. Delaney, PhD, RD, oral communication, March 2013). The CCLHO and the County Health Executives Association of California, professional organizations representing LHDs, are jointly developing a Chronic Disease Prevention Framework to advance a common chronic disease prevention agenda in California locally and statewide (24).

The Affordable Care Act’s Prevention and Public Health Fund (25) provides unprecedented resources and models for addressing chronic disease prevention and fostering new links between LHDs and medical care as well as health workforce deployment innovations to prevent chronic disease. The Phase II survey findings provide indicators that LHDs may consider as they develop community assessments and plans for chronic disease prevention activities or prepare for the public health accreditation process.

### Limitations

Although this study has many strengths, a few limitations must be noted. Most questions in Phase I were altered for Phase II, limiting our ability to compare data between time points. Altering the questions was deemed acceptable, however, because these surveys were not conceptualized to follow a pretest-posttest design. It is possible that the data aggregation methods (ie, decision rules) used to summarize multiple LHD responses into 1, for the purpose of the analysis, may have introduced a small amount of measurement error. On the other hand, incorporating multiple responses from some LHDs may have rendered the responses from those LHDs more accurate overall.

### Conclusions

Prevention-focused funding, professional development activities, continued leadership from the public health field, and community engagement are needed to ensure that LHD gains in preventing obesity and chronic disease are leveraged and sustained and that conditions underlying chronic disease are addressed. The Phase II survey findings provide a benchmark from which further increases and improvements in chronic disease prevention practice in California LHDs may be measured.
